# F51. INTEGRATED COGNITIVE REMEDIATION THERAPY PREVENTS RELAPSES IN SCHIZOPHRENIA OUTPATIENTS DURING 8-YEARS FOLLOW-UP

**DOI:** 10.1093/schbul/sby017.582

**Published:** 2018-04-01

**Authors:** Daniel Mueller, Conny Steichen, Annina R Reymond, Volker Roder

**Affiliations:** University Hospital of Psychiatry and Psychotherapy, Bern

## Abstract

**Background:**

Relapse prevention is a major aim of any treatment for schizophrenia patients. In general, recent meta-analyses showed that one third of schizophrenia patients relapse in the first year after treatment, which corresponds with rehospitalization. Since years, study data support evidence for successful relapse prevention of psycho-educative and family therapy approaches in combination with pharmacological treatment. So far little is known about the impact of Cognitive Remediation Therapy (CRT) on relapse prevention.

**Methods:**

The purpose of this RCT was to investigate whether additional CRT could prevent relapses compared to treatment as usual (TAU) defined as pharmacological and other psychosocial treatments. The CRT approach of choice was the Integrated Neurocognitive Therapy (INT) developed in our lab. INT is a group approach consisting of 4 modules including interventions on all the neuro- and social cognitive domains, defined by the MATRICS initiative, as well as educational, emotion regulation and stress reduction tasks. In this international multicenter study, a total of 156 stabilized schizophrenia outpatients, diagnosed with DSM-IV, participated. From this sample, 71 participants of two out of eight centers could be observed during a follow-up of 1, 5 and 8 years, regarding number of relapses and days of rehospitalization. Relapses were defined as increased symptoms followed by rehospitalization.

**Results:**

One year after therapy, no marked differences between INT and TAU groups in relapse rates were evident. But during 5- and 8-year follow-up, 78% and 83% of TAU patients relapsed compared to 48% and 52% of INT patients suggesting a significant benefit of INT. TAU patients suffered from more than 2 relapses after 5 years and 2.5 relapses after 8 years. In comparison, INT patients showed 0.9 relapses after 5 and 1.4 relapses after 8 years. After the 5 years follow-up there was a highly significant difference between INT and TAU, and after the 8-years a statistical tendency favoring INT could be found. Regarding the days of hospitalization, TAU patients presented a mean value of 8 days during 1 year after treatment, 90 days after 5 years and 105 days after 8 years compared to INT patients with 1.2 days after 1 year, 19 days after 5 years and 35 days after 8 years. The comparison after 1 year was close to significant, the other ones were clearly significant favoring again the INT intervention.

**Discussion:**

These data on INT intervention support evidence for an impact of CRT on relapse prevention in a 1, 5 and 8 years follow-up. However, the identification of mechanisms of change within INT treatment needs further research.

